# Clinical outcomes of helical tomotherapy for super-elderly patients with localized and locally advanced prostate cancer: comparison with patients under 80 years of age

**DOI:** 10.1093/jrr/rrv040

**Published:** 2015-08-27

**Authors:** Noriyuki Okonogi, Hiroyuki Katoh, Hidemasa Kawamura, Tomoaki Tamaki, Takuya Kaminuma, Kazutoshi Murata, Yu Ohkubo, Yosuke Takakusagi, Masahiro Onishi, Tetsuo Sekihara, Atsushi Okazaki, Takashi Nakano

**Affiliations:** 1 Department of Radiation Oncology, Gunma University Graduate School of Medicine, 3-39-22, Showa-machi, Maebashi, Gunma 371-8511, Japan; 2 Oncology Center, Hidaka Hospital, 886, Nakao-machi, Takasaki, Gunma 370-0001, Japan; 3 Department of Urology, Hidaka Hospital, 886, Nakao-machi, Takasaki, Gunma 370-0001, Japan

**Keywords:** prostate cancer, helical tomotherapy (TOMO), intensity-modulated radiotherapy (IMRT), super-elderly patients

## Abstract

We investigated the clinical outcomes of helical tomotherapy in 23 patients aged ≥80 years with localized and locally advanced prostate cancer and compared the results with data from 171 patients under 80 years. All patients received helical tomotherapy in our hospital between September 2009 and October 2012. The median follow-up periods were 35 months in the aged group and 34 months in the younger group. The median prescribed dose in helical tomotherapy was 78 Gy in 39 fractions (range, 72–78 Gy). The 3-year overall survival and biochemical relapse-free rates were 92% and 96% in the aged group and 99.4% and 97.3% in the younger group, respectively. There was no significant difference between the two groups in the biochemical relapse-free rates. The 3-year cumulative incidences of late Grade 2 or higher rectal toxicity and urinary toxicity were 13% and 4.8% in the aged group and 7.0% and 1.2% in the younger group, respectively. There was no significant difference between the aged group and the younger group in the cumulative incidence rates of rectal toxicity or urinary toxicity. No patients exhibited Grade 4 or higher toxicity, and all patients improved with conservative therapy. Helical tomotherapy in super-elderly patients with localized and locally advanced prostate cancer had good biochemical control rates without severe late toxicity. Definitive helical tomotherapy may be the treatment of choice for patients with localized and locally advanced prostate cancer, even in those older than 80 years of age.

## INTRODUCTION

Prostate cancer is one of the most common malignancies in the USA [1], Europe [2] and Japan [3]. The incidence of prostate cancer is increasing among the aging population in Japan [3]. The US National Cancer Institute database lists a median age at diagnosis of 68 years, and 71.2% of deaths due to prostate cancer occur in men aged over 75 years [4]. With the dramatic aging of populations and increasing life expectancy (LE), especially in developed countries [5–7], further increases in the number of patients with prostate cancer are expected.

Guidelines for the management of elderly patients (usually defined as aged 65 or older) with prostate cancer have been established based on accumulating clinical data [8–11]. The National Comprehensive Cancer Network (NCCN) guidelines emphasize that estimating LE is important when choosing the therapeutic strategy for localized and locally advanced prostate cancer [8]. Definitive radiation therapy is considered for intermediate- and high-risk groups of prostate cancer patients with LE of <10 years [8]. Alibhai *et al.* [9] developed a Markov state transition model to evaluate LE and quality-adjusted LE (QALE) in men older than 65 years with localized prostate cancer. They concluded that radical prostatectomy and definitive radiation therapy improved the LE and QALE up to age 75 years for moderately differentiated tumors (Gleason 5–7), and curative therapy improved the LE and QALE up to age 80 years for poorly differentiated tumors (Gleason 8–10).

Recent studies show that elderly men are more likely to be diagnosed with higher-risk prostate cancer than younger men [4, 12]. As the elderly population grows, the number of patients with prostate cancer who require definitive radiation therapy is expected to increase accordingly. However, the Cancer of the Prostate Strategic Urologic Research Endeavor database indicates that men aged over 75 years were more likely to be treated conservatively and to die from prostate cancer [12, 13]. One reason that the appropriate treatment is not selected for elderly prostate cancer patients may be the lack of data on the clinical outcomes of definitive therapy for elderly patients with prostate cancer.

Hence, clinical outcomes in super-elderly patients (80 years and older) with localized and locally advanced prostate cancer treated with intensity-modulated radiation therapy (IMRT) in our hospital were investigated. The data were compared with those from prostate cancer patients under the age of 80 years. In addition, the correlation between the dosimetric parameters and late toxicity was analyzed.

## MATERIALS AND METHODS

### Patient characteristics

A total of 23 patients aged 80 years or older with T1c–T4 non-metastatic prostate cancer (according to the International Union Against Cancer TNM Classification of Malignant Tumors, 7th edition) were treated with IMRT in our hospital between September 2009 and October 2012. All patients were included in the present study. To evaluate the clinical outcomes in super-elderly patients who were 80 years old or above, data were compared with those from 171 patients under the age of 80 years with intermediate-risk, high-risk and castration-resistant prostate cancer (CRPC) who were treated around the same time in our hospital. The institutional review board of our hospital approved this study.

Table 1 shows the patient characteristics in the present study. The median ages were 81 years old (range, 80–87) in patients aged 80 years and above (the aged group) and 71 years old (range, 46–79 years) in patients under the age of 80 years (the younger group). All patients were classified using the D'Amico risk group classification [14]. Eight patients were classified as intermediate-risk and 15 as high-risk in the aged group; 59 patients were classified as intermediate-risk and 112 patients were classified as high-risk in the younger group at the time of diagnosis. Eight patients in the aged group and five patients in the younger group were diagnosed with CRPC before IMRT initiation because of a continuous increase in the serum levels of prostate-specific antigen (PSA), despite the castrated levels of serum testosterone (<50 ng/dl). Therefore, five patients were intermediate-risk, 10 were high-risk, and eight had CRPC in the aged group, and 58 patients were intermediate-risk, 108 patients were high-risk, and five patients had CRPC in the younger group at the start of IMRT.

**Table 1.** Patient characteristics

Characteristics	Patients aged ≥80 years (*n* = 23)		Patients aged <80 years (*n* = 171)		
	Number	%	Number	%	*P* value
Age (years)	80–87	81 (median)	46–79	71 (median)	
ECOG performance status					0.47
0	19	83	135	79	
1	4	17	36	21	
≥ 2	0	0	0	0	
Pretreatment PSA (ng/ml)	4.9–79.6	10.0 (median)	3.1–512.0	10.2 (median)	0.71
≤ 10	12	52	82	48	
10–20	6	26	43	25	
≥ 20	5	22	46	27	
Gleason score					0.15
≤ 6	3	13	11	6	
7	8	35	94	55	
≥ 8	12	52	66	39	
Tumor stage					0.91
T1c–T2a	9	39	71	41	
T2b	4	17	25	15	
T2c–T4	10	44	75	44	
Risk group^a^					<0.01
Intermediate	5	22	58	34	
High	10	43	108	63	
CRPC	8	35	5	3	
Diabetes					0.72
Yes	4	17	30	18	
No	19	83	141	82	
Anticoagulants					0.53
Yes	6	26	35	20	
No	17	74	136	80	

^a^The risk group was defined at the time of starting helical tomotherapy. ECOG = Eastern cooperative oncology group, PSA = prostate-specific antigen, CRPC = castration-resistant prostate cancer.

The median periods from the start of androgen deprivation therapy (ADT) to diagnosis with CRPC were 73 months (range, 14–138 months) in the aged group and 37 months (range, 22–61 months) in the younger group. The median follow-up periods after IMRT were 35 months (range, 19–53 months) in the aged group and 34 months (range, 11–61 months) in the younger group. Written informed consent was obtained from all patients prior to treatment.

Thoraco–abdominal computed tomography (CT, Aquilion LB, Toshiba Medical, Otahara, Japan) and pelvic magnetic resonance images (MRIs, Vantage Titan and Pianissimo, Toshiba Medical, Otahara, Japan) were performed for staging before the start of IMRT in all patients. Whole-body bone scintigraphy was also performed before IMRT initiation for patients with high-risk prostate cancer and CRPC.

### IMRT using helical tomotherapy

IMRT is a powerful tool for improving the quality of the delivered dose distribution in external beam radiation therapy, and this therapy reduces late rectal toxicity in high-dose external beam radiation therapy for prostate cancer [15]. The TomoTherapy Hi-Art system (Accuray Inc., Sunnyvale, CA, USA) is a radiation delivery system that combines dynamic IMRT and an image-guided radiation therapy system [16, 17]. All patients with prostate cancer were treated with IMRT using helical tomotherapy (TOMO) in the present study.

### CT simulation and target delineation

Patients were placed in the supine position with a universal fixation device (ESFORM, Engineering System Co., Nagasaki, Japan) to immobilize the lower legs and reduce set-up error. Axial images (3-mm slices) were acquired using a 16-row multi-detector CT (Aquilion LB, Toshiba Medical, Otahara, Japan) for planning CT. Pelvic MRI at 3-mm thickness was performed, and T2-weighted images were fused to the planning CT images to delineate target volumes. Contouring of target volumes and normal structures was performed on the Focal treatment planning system, version 4.3.1 (Focal Eindhoven, Netherlands).

The clinical target volume (CTV) was defined as the entire prostate and proximal seminal vesicles. The CTV was defined as the entire prostate and all of the seminal vesicles in patients with T3b disease. The planning target volume (PTV) encompassed the CTV with a 5-mm margin in the bilateral, craniocaudal and anterior directions, and there was a 3-mm margin in the posterior direction. The rectum was contoured as a solid structure defined by the outer wall from the anal canal to the rectosigmoid junction. The bladder was also contoured as a solid structure defined by the outer wall. Two or more radiation oncologists examined all contoured structures to provide consistency in defining the target volumes. The contours created in the treatment-planning system were transferred to the TomoTherapy Hi-Art treatment-planning system, v4.0, and the TOMO plans were generated.

### Planning of TOMO

The PTVs primarily received 78 Gy in 39 fractions in the present study. Three cases in which the intestines were close to the PTV received 72 Gy in 36 fractions to reduce toxicity. The prescription dose was defined as the minimum dose delivered to 95% of the PTV (D95%). The maximum tolerated dose in the PTV was limited to <105% of the prescription dose. Rectal dose–volume constraints limited V65 Gy (percentage of the rectum volume receiving at least 65 Gy) to ≤17%, V40 Gy to ≤35% and V22 Gy to ≤60%. The bladder dose–volume constraints limited V65 Gy to ≤25% and V40 Gy to ≤50%.

The appropriate dose constraints were implemented for inverse planning procedures. The following treatment parameters were used to generate the TOMO plans: field width of 2.48 cm, modulation factor of 2.0, and pitch of 0.287. To reduce the dose to critical organs, protective measures such as rectal blocking structures were appropriately added in the TOMO planning. All optimization procedures were performed until the both the PTV and other organs' dose–volume constraints were achieved. The entire optimization was restarted when constraints were violated. A fine grid (2.7 mm × 2.7 mm) was used for the final calculation process after all constraints were satisfied.

### Daily treatments of TOMO

Image-guided radiation therapy was performed daily in all patients. Acquired CT images using mega voltage CT (MVCT) were superimposed onto the treatment plans. The patient's position was adjusted according to prostate matching before each treatment. Patients had a tube inserted or were encouraged to defecate when their rectums were dilated for daily MVCT and were re-examined on MVCT.

### Androgen deprivation therapy

Urologists administered ADT, including medical or surgical castration, to all 23 patients of the aged group and all 171 patients of the younger group. Among the patients aged 80 years and above, the median period of ADT before or concomitant with TOMO was 17 months (range, 5–141 months) for all 23 patients; the median periods of ADT before TOMO were 7 months (range, 5–31 months) for the intermediate-risk group, 11 months (range, 6–46 months) for the high-risk group, and 84 months (range, 45–141 months) for the CRPC group. The median periods of ADT after TOMO were 25 months (range, 10–45 months) for the intermediate-risk group and 31 months (range, 0–46 months) for the high-risk group. Fourteen patients received ADT at the last follow-up. Among the patients under the age of 80 years, the median period of ADT before or concomitant with TOMO was 12 months (range, 5–63 months) for all 171 patients; the median periods of ADT before TOMO were 7 months (range, 5–13 months) for the intermediate-risk group, 10 months (range, 5–29 months) for the high-risk group, and 39 months (range, 24–63 months) for the CRPC group. The median periods of ADT after TOMO were 20 months (range, 0–41 months) for the intermediate-risk group and 32 months (range, 6–55 months) for the high-risk group. A total of 106 patients received ADT at the last follow-up.

### Follow-up after TOMO and data collection

A urologist and a radiation oncologist conducted patient follow-ups at 3-month intervals for the first 3 years after TOMO and at intervals of 3–6 months thereafter. PSA values were evaluated in each follow-up examination. Biochemical relapse was defined as the nadir PSA level plus 2 ng/ml. CT images were collected once per year after TOMO to assess any metastatic progression. Late toxicity was evaluated according to the Common Terminology Criteria for Adverse Events, version 3.0. Patients with suspected rectal bleeding underwent endoscopic examinations, and late rectal toxicity was confirmed.

For patients aged 80 years and above, dose–volume histogram (DVH) parameters were collected from each treatment plan on the TomoTherapy Hi-Art treatment-planning system to clarify the effect of late toxicity. Patients were divided into two groups: those with any late toxicity and patients without late toxicity. We compared the DVH parameters and complications, such as diabetes mellitus and conditions requiring the use of anti-coagulation therapy, in these two groups. The present study analyzed the PTV volume, rectal volume, maximum rectal dose, and minimal radiation doses for the most irradiated rectal volumes of 2 cc (Rectum D2 cc), V70 Gy, V60 Gy, V50 Gy, V40 Gy and V30 Gy to assess effects on late rectal toxicity. Similarly, the PTV volume, bladder volume, maximum bladder dose, Bladder D2 cc, V70 Gy, V60 Gy, V50 Gy, V40 Gy and V30 Gy were analyzed to assess effects on late urinary toxicity.

### Statistical analyses

The overall survival time, biochemical relapse-free time, and cumulative occurrence rates of late toxicity were estimated using the Kaplan–Meier method. Comparisons of the overall survival time, biochemical relapse-free time, and cumulative occurrence rates of late toxicity were assessed by Log-rank analysis. Comparisons of the clinical and dosimetric factors between the two groups based on toxicity were assessed using Fisher's exact test, the Wilcoxon signed-rank test, and Welch's unpaired *t*-test. Multivariable logistic regression analysis was performed for factors that previously appeared to be associated with the risk of late rectal toxicity using the *t*-test (*P* < 0.05). Significant differences were assessed using two-sided tests with *P* < 0.05. All statistical analyses were performed using Statistical Package for the Social Sciences (SPSS) software, version 16.0 (SPSS, Chicago, IL, USA).

## RESULTS

### Overall survival and biochemical relapse-free rates

**Fig. 1.**
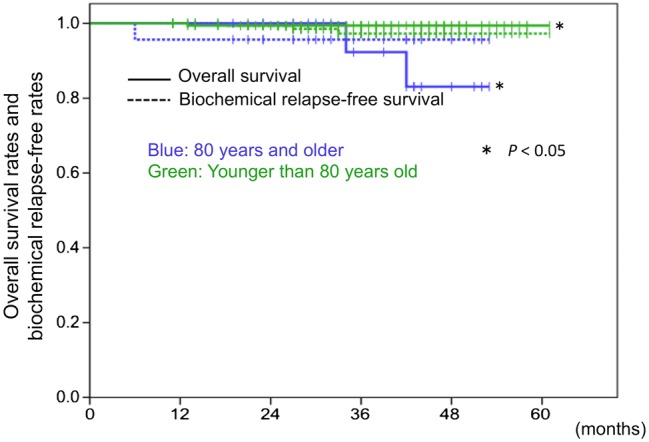
Overall survival rates and biochemical relapse-free rates after TOMO. Solid lines show the overall survival rates, and dashed lines indicate the biochemical relapse-free rates after TOMO. Blue lines depict the rates for patients 80 years and older, and green lines illustrate rates for patients younger than 80 years old. There was a significant difference in the overall survival rates.

Figure 1 shows the overall survival rates and biochemical relapse-free rates. In the cohort of patients aged 80 years and older, the 3-year overall survival and biochemical relapse-free rates were 92% and 96%, respectively. Two patients had died by the final follow-up. One patient had exhibited biochemical relapse at 6 months and had died of metastatic prostate cancer at 34 months after TOMO initiation, and the other patient died of gastric cancer at 42 months after the initiation of TOMO, with no evidence of prostate cancer recurrence. The patient who died of metastatic prostate cancer after TOMO was in the high-risk group. All eight CRPC patients had no evidence of disease at the final follow-up. In the cohort of patients younger than 80 years of age, the 3-year overall survival and biochemical relapse-free rates were 99.4% and 97.3%, respectively. There was a significant difference between the aged group and the younger group in the overall survival rates (*P* < 0.014). There was no significant difference between the two groups in the biochemical relapse-free rates.

### Late toxicity

Table 2 shows the maximal late toxicities in the present study. No Grade 4 or 5 late toxicity was observed in any of the patients. Of the patients aged 80 years and older, Grade 3 late toxicity was observed in one patient, who developed massive rectal bleeding and required a blood transfusion 22 months after the initiation of TOMO. Grade 2 late toxicity was observed in three patients: two patients with rectal bleeding and one patient with urinary retention. None of the patients with Grade 2 or 3 toxicity required surgical intervention (e.g. laser coagulation), and these patients improved with conservative therapy (e.g. oral drug administration). Grade 1 late toxicity was observed in five patients; four patients had slight rectal bleeding and four had urinary frequency. Some patients exhibited both rectal and urinary events. No late toxicity was observed in the remaining 14 patients. Of the patients younger than 80 years, Grade 3 late toxicity was observed in three patients, and Grade 2 late toxicity was observed in 10 patients. None of these patients required surgical intervention.

**Table 2.** Maximal late toxicities in all cases

	CTCAE v3.0 Grade						
	0	1	2	3	4	5	Total
Patients aged ≥80 years							
Rectal toxicity	16	4	2	1	0	0	23
Urinary toxicity	18	4	1	0	0	0	23
Total^a^	14	5^a^	3	1	0	0	23
Patients aged <80 years							
Rectal toxicity	128	32	8	3	0	0	171
Urinary toxicity	125	44	2	0	0	0	171
Total^a^	96	62^a^	10	3	0	0	171

^a^Some patients showed both rectal and urinary events. CTCAE v3.0 = The Common Terminology Criteria for Adverse Events, version 3.0.

**Fig. 2.**
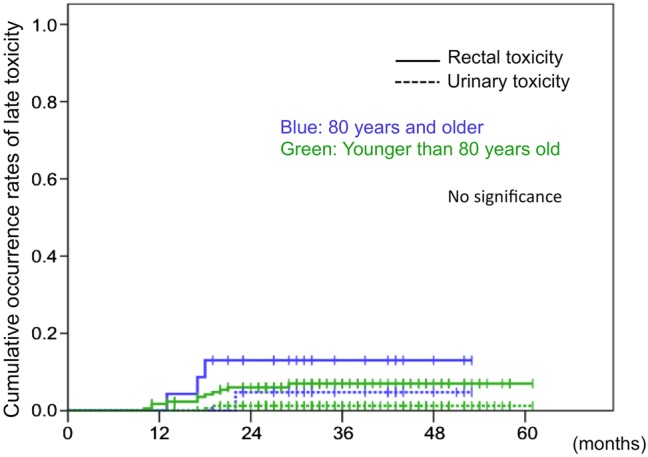
Cumulative occurrence rates of late Grade 2 or higher rectal and urinary toxicities after TOMO. The solid lines show the cumulative occurrence rates of rectal toxicity, and dashed lines indicate the cumulative occurrence rates of urinary toxicity. Blue lines illustrate rates for patients 80 years and older, and green lines show the rates for patients younger than 80 years old. There was no significant difference in the cumulative incidence rate or rectal or urinary toxicity.

Figure 2 shows the cumulative occurrence rates of developing late Grade 2 or higher rectal or urinary toxicity after TOMO. In the aged group, the 3-year cumulative incidences of rectal and urinary toxicity were 13% and 4.8%, respectively. In the younger group, the 3-year cumulative incidence rates of rectal and urinary toxicity were 7.0% and 1.2%, respectively. There was no significant difference between the aged group and the younger group in either cumulative incidence rates of rectal toxicity or urinary toxicity.

### The effects of patient characteristics and DVH parameters on late rectal toxicity

Table 3 shows the effects of patient characteristics and DVH parameters on late rectal toxicity in patients aged 80 years and older. Patients with late rectal toxicity had significantly higher mean rectal values of V40 Gy, V50 Gy, V60 Gy and V70 Gy. The most significant difference was observed for V70 Gy (*P* < 0.005). Multivariable logistic regression analysis for the factors that were associated with the risk of late rectal toxicity using the *t*-test indicated that none of the factors was significantly correlated in the analysis (data not shown).

**Table 3.** Patient characteristics and mean values of the rectal dosimetric parameters in patients aged 80 years and older

Parameters	CTCAE v3.0 Grade		
	Grade 0 (*n* = 16)	Grade 1–3 (*n* = 7)	*P* value
Age (years)	82.1 ± 2.0	81.6 ± 1.9	n.s.
Rectal volume (cm^3^)	64.8 ± 17.7	51.3 ± 14.2	n.s.
PTV (cm^3^)	68.5 ± 28.3	66.0 ± 18.5	n.s.
Diabetes no. (%)	3 (18)	1 (14)	n.s.
Anticoagulants no. (%)	6 (38)	0 (0)	n.s.
Rectum V 30 (%)	27.8 ± 12.1	33.6 ± 7.6	n.s.
V 40 (%)	17.4 ± 9.9	27.1 ± 7.0	0.016
V 50 (%)	13.0 ± 6.8	20.6 ± 6.4	0.025
V 60 (%)	9.3 ± 5.2	15.1 ± 4.9	0.017
V 70 (%)	4.9 ± 3.3	9.8 ± 2.9	0.003
D 2 cc (Gy)	74.3 ± 5.2	77.4 ± 3.0	n.s.
D max (Gy)	79.2 ± 2.4	80.7 ± 0.7	n.s.

Age, rectal volume, PTV, and dose-volume histogram parameters are represented as the means ± standard deviation. CTCAE v3.0 = The Common Terminology Criteria for Adverse Events version 3.0, PTV = planning target volume, no. = number, V dose = the percentage of the rectum at least covered by each dose, n.s. = not significant.

### The effects of patient characteristics and DVH parameters on late urinary toxicity

Table 4 shows the effects of patient characteristics and DVH parameters on late urinary toxicity in the cohort of patients aged 80 years and older. There were no significant differences between the patients with and without late toxicity in any patient characteristics or DVH parameters.

**Table 4.** Patient characteristics and mean values of the urinary dosimetric parameters in patients aged 80 years and older

Parameters	CTCAE v3.0 Grade		
	Grade 0 (*n* = 18)	Grade 1–2 (*n* = 5)	*P* value
Age (years)	81.9 ± 1.9	81.8 ± 1.9	n.s.
Bladder volume (cm^3^)	282.2 ± 163.2	199.8 ± 62.1	n.s.
PTV volume (cm^3^)	66.0 ± 19.7	55.6 ± 5.8	n.s.
Diabetes no. (%)	4 (22)	0 (0)	n.s.
Anticoagulant no. (%)	5 (28)	1 (20)	n.s.
Bladder V 30 (%)	42.6 ± 20.1	43.5 ± 12.9	n.s.
V 40 (%)	31.7 ± 15.3	32.3 ± 9.8	n.s.
V 50 (%)	23.4 ± 11.6	23.7 ± 7.2	n.s.
V 60 (%)	16.8 ± 8.5	16.9 ± 5.0	n.s.
V 70 (%)	10.8 ± 5.4	10.9 ± 3.6	n.s.
D 2 cc (Gy)	78.9 ± 2.3	79.5 ± 0.3	n.s.
D max (Gy)	79.9 ± 2.5	80.1 ± 0.3	n.s.

Age, bladder volume, PTV and dose–volume histogram parameters are represented as the means ± standard deviation. CTCAE v3.0 = The Common Terminology Criteria for Adverse Events version 3.0, PTV = planning target volume, no. = number, V dose = the percentage of the rectum at least covered by each dose, n.s. = not significant.

## DISCUSSION

In the patients aged 80 years and older, only one patient died of prostate cancer metastases 34 months after TOMO, and all other patients were alive without recurrence after TOMO at the final follow-up (median follow-up time: 35 months). There was a significant difference in the overall survival rates between the cohort of patients younger than 80 years old and those who were 80 years and older. However, this difference was due to the death of two patients in the aged group, one of whom died from gastric cancer. It is unlikely that the difference in ages resulted in a difference in the therapeutic effect of TOMO. Additionally, the 3-year cumulative incidences of late Grade 2 or higher rectal toxicity and urinary toxicity in the present study were 13% and 4.8%, and no patients with Grade 4 or higher toxicity were observed in the present study. The incidences of toxicity were not significantly different in the older group from those of patients younger than 80 years old. Additionally, these incidences of toxicity were similar to those reported in previous studies of high-dose IMRT for prostate cancer, most of which were undertaken in patients aged 80 years or younger [18–23]. These findings indicate that TOMO for patients who are 80 years and older with localized and locally advanced prostate cancer may be acceptable and beneficial. The burden of prostate cancer is expected to increase dramatically with the exponential aging of populations and increasing LE, especially in developed countries [5–7]. Definitive radiotherapy based on the careful consideration of an individual's prostate cancer risk profile should be considered, even for patients over 80 years of age.

Recently, Tomita *et al.* [24] reported a relationship between the dosimetric parameters and late rectal toxicity for localized prostate cancer patients treated with TOMO. They demonstrated that rectal V60 Gy and V70 Gy, and the maximum dose to the rectum, were significantly higher in the ≥ Grade 2 toxicity group than the ≤ Grade 1 toxicity group. There was a similar trend in that patients with late rectal toxicity had significantly higher mean values of rectal V40 Gy, V50 Gy, V60 Gy and V70 Gy. Hence, we recommend the use of stringent dose–volume constraints in middle to high doses to the rectum in TOMO for prostate cancer to reduce rectal toxicity. No significant correlation was observed between the dosimetric parameters of the bladder and late urinary toxicity in the present study.

More than one-third (8 of 23) of the patients aged 80 years or older in the present study had CRPC, probably because most super-elderly patients were administered ADT without local treatment as the primary treatment in practice [25, 26]. After the disease developed into CRPC, such a patient would be recommended to receive radiotherapy, such as TOMO. However, several recent reports have demonstrated inadequate efficacy of radiotherapy for CRPC, and the biochemical relapse-free survival rates in these reports were not satisfactory [27, 28]. The use of this conventional radiotherapy technique, which used lower prescribed doses than the present study, might explain these unsatisfactory outcomes. In fact, the use of high-dose radiotherapy with TOMO in all eight patients with CRPC in the present study eliminated biochemical recurrence until the final follow-up. These findings indicate that optimized high-dose radiotherapy may achieve better outcomes, even for CRPC.

The most recently released NCCN guidelines do not define established treatment strategies for CRPC patients without signs of lymph node or distant metastases [8]. Androgen receptor status remains positive in most patients who develop CRPC, and secondary hormone therapy such as anti-androgen, ketoconazole, steroids, diethylstilbestrol, or other estrogens, are other options for CRPC treatment [29, 30]. However, no randomized clinical trial has demonstrated benefits in terms of survival rates. Moreover, secondary hormone therapy is often not used for super-elderly patients in clinical practice due to an increased risk of adverse effects, such as diabetes, cardiovascular complications and osteoporosis. The present study indicated that TOMO achieved advantageous outcomes with low toxicities in super-elderly patients with CRPC. Therefore, high-dose IMRT using TOMO is one option for the salvage treatment of super-elderly patients with node-negative, localized CRPC.

The present study has several limitations, including the retrospective nature of the study, the small number of patients analyzed, and the short follow-up period. In addition, all patients who received IMRT with TOMO exhibited good performance status without severe comorbidities. Rockwood *et al.* [31] reported that senior adult patients who were dependent in terms of daily living activities had a shorter survival. Furthermore, Guzzo *et al.* [32] reported that the non-prostate cancer–specific mortality rate of patients with localized prostate cancer treated with radical prostatectomy was higher than the prostate cancer–specific mortality rate, and the Charlson index [33], which evaluates comorbidities, was the strongest predictor of death from causes other than prostate cancer. Taken together, when we treat super-elderly patients with prostate cancer using any treatment, including TOMO, it is necessary to thoroughly examine the suitability of the treatment through assessing the performance status, comorbidities and risk of prostate cancer.

Active surveillance is considered the best option for patients with low-risk prostate cancer [8]. In fact, patients with low-risk prostate cancer were not included in this study. The NCCN guidelines recommend starting treatment in most patients who have a Gleason grade of 4 or 5 on repeat biopsy, cancer in a larger number or greater extent of prostate biopsies, or a PSA doubling time of less than 3 years [8]. ADT might be commonly used as a treatment option for patients with prostate cancer that has progressed after active surveillance in the clinical setting, especially in the elderly. However, Lu-Yao *et al.* [34] reported that there was no survival benefit in patients receiving ADT compared with observation alone in an analysis of 19 271 elderly patients who did not receive definitive local therapy for clinical stage T1–T2 prostate cancer. ADT should not be used in routine practice for elderly patients with low-risk prostate cancer. Additionally, some studies have reported an association between ADT and an increased risk of diabetes [35], cardiovascular morbidity [36], and bone fractures [37]. These ADT-related adverse effects could be fatal in super-elderly patients. Although ADT is generally considered to be effective in combination with radiation therapy for patients with intermediate- and high-risk prostate cancer, further research is required to identify the suitable period of ADT for super-elderly patients with prostate cancer.

Our findings show that TOMO may be beneficial in patients who are 80 years and older with localized and locally advanced prostate cancer. Further follow-up is required to determine long-term outcomes.

## FUNDING

This work was supported by the Japan Society for the Promotion of Science, Grant-in-Aid for Exploratory Research [JSPS KAKENHI grant number 25670527]. Funding to pay the open access publication charges for this article was also provided by this grant.
